# Effect of anticoagulant and platelet inhibition on the risk of bacteremia among patients with acute pyelonephritis: a retrospective cohort study

**DOI:** 10.1186/s12879-022-07474-4

**Published:** 2022-05-31

**Authors:** Svava E. Steiner, Gustaf Edgren, Keira Melican, Agneta Richter-Dahlfors, Annelie Brauner

**Affiliations:** 1 AIMES - Center for the Advancement of Integrated Medical and Engineering Sciences at Karolinska Institutet and KTH Royal Institute of Technology, Stockholm, Sweden; 2 Department of Neuroscience, Karolinska Institutet, 171 77 Stockholm, Sweden; 3 Department of Medicine Solna, Clinical Epidemiology Division, Karolinska Institutet, 171 77 Stockholm, Sweden; 4 Department of Cardiology, Södersjukhuset, Stockholm, Sweden; 5 Department of Microbiology, Tumor and Cell Biology, Karolinska Institutet, 171 77 Stockholm, Sweden; 6 Division of Clinical Microbiology, Karolinska University Hospital, 171 76 Stockholm, Sweden

**Keywords:** Acute kidney injury, Anticoagulants, Bacteremia, Platelet aggregation inhibitors, Urinary tract infection

## Abstract

**Background:**

An increasing number of patients are being prescribed anticoagulants and platelet inhibitors (antithrombotic treatment). Basic research has suggested an association between antithrombotic treatment and bacteremia during kidney infection. Here, we investigated the association between antithrombotic treatment, bacteremia and acute kidney injury in patients with acute pyelonephritis.

**Methods:**

A retrospective cohort study was conducted in a large university hospital in Sweden. Data were retrieved from electronic medical records for adult patients with acute pyelonephritis in 2016. The main outcome was bacteremia and secondary outcome acute kidney injury. Odds ratios (ORs) with 95% confidence intervals (CIs) were estimated through multiple logistic regression. Treatment with different groups of antithrombotic agents were compared to no antithrombotic treatment.

**Results:**

1814 patients with acute pyelonephritis were included, in whom bacteremia developed in 336 (18.5%). Low-molecular-weight heparin (LMWH) at prophylactic doses was associated with a lower risk of bacteremia, compared to no antithrombotic treatment (OR 0.5; 95% CI 0.3–0.7). Other antithrombotic treatments were not associated with a risk of bacteremia. Additionally, patients with prophylactic doses of LMWH had a lower risk of acute kidney injury (OR 0.5; 95% CI 0.3–0.8).

**Conclusions:**

We found no association between antithrombotic treatment and an increased risk of bacteremia during acute pyelonephritis. Conversely, patients with prophylactic doses of LMWH had a slightly reduced risk of bacteremia. LMWH at prophylactic doses was also associated with a lower risk of acute kidney injury. Our results suggest that it is safe to continue antithrombotic treatment during acute pyelonephritis, in regards to bacteremia and acute kidney injury risk.

**Supplementary Information:**

The online version contains supplementary material available at 10.1186/s12879-022-07474-4.

## Background

Acute pyelonephritis is a common bacterial infection, often caused by Gram-negative bacteria, predominantly *Escherichia coli*. The infection can sometimes be associated with complications such as bacteremia and urosepsis [1], as well as acute kidney injury [2]. Considering the relatively high frequency of bacteremia, combined with high reported mortality rates [3], it is important to identify potential risk factors for severe outcomes.

During bacterial infection, there is an interplay between inflammation and coagulation, with thrombosis playing an important role in innate immunity [4–6]. Many studies have investigated anticoagulants as potential adjunctive treatments of sepsis [7–12]. Little is known, however, about the role of coagulation during localized bacterial infections, prior to systemic spread of bacteria. In rodents, vascular thrombus formation has been observed during experimental kidney infection [13–17]. We previously visualized clot formation in peritubular capillaries following a localized kidney tubule infection [14–16]. This clot formation appeared to be protective against urosepsis, as anticoagulant treatment delayed the initiation of clotting and resulted in systemic spread of bacteria [14]. While these results suggested a role of blood clots in preventing bacteremia in rodents, it implied that patients with antithrombotic treatment may be at higher risk of developing urosepsis during acute pyelonephritis.

The number of patients being prescribed different types of antithrombotic drugs (both anticoagulants and platelet inhibitors) is increasing worldwide. This is partially due to the introduction of routine screenings for atrial fibrillations along with an aging population [18]. Considering both the relatively high incidence of acute pyelonephritis, and the high numbers of patients on antithrombotic treatment, it is important to evaluate the possible impact of such treatment on the risk of developing bacteremia for patients with urinary tract infections. In this study, we investigated if antithrombotic treatment is associated with an increased risk of bacteremia in patients with acute pyelonephritis. While coagulation surrounding infected areas might potentially prevent bacteremia, it may also result in increased renal injury, which is important to investigate from a clinical perspective. Therefore, we also studied the association between antithrombotic treatment and risk of acute kidney injury among patients with acute pyelonephritis.

## Methods

### Study design and setting

The overall aim of the study was to investigate if antithrombotic treatment is associated with an increased risk of bacteremia or acute kidney injury in patients with acute pyelonephritis. We conducted a retrospective cohort study using data from the electronic medical record system at Karolinska University Hospital in Stockholm, Sweden. The study was based on all inpatients diagnosed with acute pyelonephritis, from January 1 to December 31, 2016.

### Data sources and participants

We extracted data from two sources. Data on all bacterial cultures were extracted from the laboratory information management system kept by Karolinska University Laboratory, where all microbiological analyses for the hospital are performed. Using unique national registration numbers, assigned to all inhabitants of Sweden, data were linked to the hospital electronic medical record system. This provided data on demographics (age and sex), antithrombotic use (see Additional file 1: Table S1), vital signs (heart rate, body temperature, blood pressures, peripheral oxygen saturation, respiratory rate, and Glasgow Coma Scale [GCS]), objective physical findings (body mass index [BMI]), laboratory findings, information regarding the hospital visit (ward, duration of hospital stay, reason for hospitalization), and comorbidities.

**Fig. 1:**
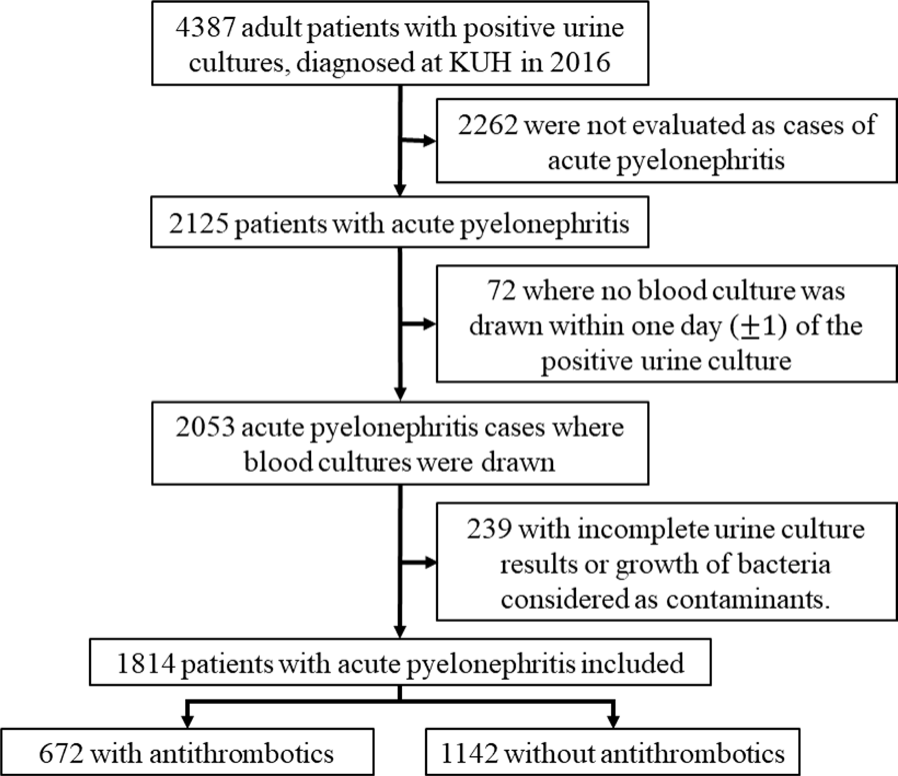
Flowchart of the study population. KUH: Karolinska University Hospital

From the linked hospital record data, we identified all adult patients with suspected acute pyelonephritis (defined as growth of bacteria in urine combined with body temperature ≥ 38 °C or a diagnosis of pyelonephritis, as defined in Additional file 1: Table S2). If a patient had more than one episode of acute pyelonephritis during the study period, only the first episode was included. Urine samples with growth of bacteria considered as normal microbiota or contamination were excluded. To allow the ascertainment of the occurrence of bacteremia, the study population was further restricted to patients with at least 1 blood culture drawn within one day of the urine culture. See Fig. 1 for study profile. For our secondary analysis, investigating the risk of acute kidney injury, we included all patients with acute pyelonephritis, and with available data on the outcome measurement (definition detailed below). See Additional file 1: Fig. S1 for study profile of this sub-cohort.

### Definitions

Antithrombotic treatment was defined as having a recorded continuous use of drugs with Anatomic Therapeutic Chemical (ATC) classification codes starting with “B01” from at least one day prior to the acute pyelonephritis episode and at least until the day of the positive urine culture (see Additional file 1: Table S1 for all included antithrombotic treatments). Patients without recorded continuous use of antithrombotics during the study period were considered unexposed. For our main analyses, we stratified antithrombotic treatment into five categories. If a patient was prescribed more than one antithrombotic treatment, only one treatment was chosen for each patient in the following order: (i) low-molecular-weight heparin (LMWH) at therapeutic doses, (ii) low-molecular-weight heparin (LMWH) at prophylactic doses, (iii) other anticoagulants (including Warfarin and direct oral anticoagulants [DOACs]), (iv) platelet-inhibitors except acetylsalicylic acid, and (v) acetylsalicylic acid (for details, see Additional file 1: Table S2). Comorbid conditions were identified and classified using ICD-10 coding. The definitions used for comorbidities of interest or characteristics important for cohort definition are listed in Additional file 1: Table S2.

### Outcomes

The main outcome was bacteremia, defined as at least one positive blood culture drawn within one day of the urine culture, with growth of the same bacterial strain in blood and urine. If a blood culture did not show any growth of bacteria or was discordant to the urine culture, it was considered negative. In secondary analyses, we used acute kidney injury as outcome. This was defined as either a discharge diagnosis of acute kidney injury, or as an increase of serum creatinine by ≥ 26.4 µmol/L, or a relative increase of serum creatinine of ≥ 50% within 48 h from baseline at the time of the urine culture, according to Acute Kidney Injury Network classification stage 1 or higher [19]. Baseline serum creatinine levels were defined as the latest value taken more than one day before the acute pyelonephritis episode.

### Statistical analysis

Descriptive statistics were reported as absolute numbers and percentage for categorical variables, and median with interquartile range for age. Odds ratios (OR) of bacteremia, and acute kidney injury, comparing antithrombotic treatment to no antithrombotic treatment at the time of the acute pyelonephritis episode, were calculated using multiple logistic regression. Adjusted ORs, with 95% confidence intervals (CIs) were reported. In addition to terms for antithrombotic treatment, the logistic regression models included terms for age, sex, BMI, and the following comorbidities: malignancy, diabetes, hypertension, chronic renal failure, chronic heart failure, chronic liver disease, cardiovascular disease, thromboembolism and coagulopathy. Variables were chosen a priori based on clinical judgment. Age and BMI were included as categorical variables (see Additional file 1: Table S2), while all other variables were treated as binary categorical predictors. For BMI, missing data was included as a separate category. All statistical analyses were performed using SAS version 9.4 (SAS Institute, Cary, NC).

## Results

### Study population and characteristics

In total 4387 patients with positive urine cultures were considered for inclusion (Fig. 1). Of these 2125 were found to have acute pyelonephritis. Blood cultures were taken from 2053 of these patients. 1814 patients with acute pyelonephritis remained in the final cohort after exclusion of cases where urine culture revealed growth of microorganisms interpreted as clinically not significant or with inconclusive results. In this cohort, 672 (37.0%) were on some form of anticoagulant or platelet inhibitor (antithrombotic) treatment at the time of the urine culture.

Patients with antithrombotic treatment were more often male, older, had a higher BMI, and were more likely to have a history of diabetes, malignancy, hypertension, chronic renal failure, congestive heart failure, tachycardia arrhythmia (including atrial fibrillation), cardiovascular disease and thromboembolism (Table 1). In the full cohort, the most common bacterial strain found in urine was *E. coli* (n = 1078), followed by *Enterococcus* species (n = 281), and *Klebsiella pneumoniae* (n = 192) (Additional file 1: Table S3). However, *E. coli* infection was less common among patients with antithrombotic treatment, who more often had polymicrobial infections. While an indwelling urinary catheter was more common among patients with antithrombotic treatment, we found no differences in levels of C-reactive protein (CRP) or white blood cell (WBC) count between patients with or without antithrombotic treatment (Table 1). Among the 1814 patients with acute pyelonephritis, 336 (18.5%) had bacteremia, and *E. coli* was the most common species found in blood cultures (n = 221), followed by *Klebsiella pneumoniae* (n = 41) (Additional file 1: Table S4). Anticoagulants were prescribed to 396, and platelet inhibitors in 328 of the patients with acute pyelonephritis. LMWH was prescribed at therapeutic doses to 95 patients, and at prophylactic doses to 214 patients. Non-LMWH anticoagulants (including Warfarin and DOAC) were prescribed to 87 patients, while acetylsalicylic acid was prescribed to 213 and non- acetylsalicylic acid platelet inhibitors to 63 patients.

**Table 1:** Clinical characteristics of patients in our cohort

	Cohort	Antithrombotic treatment	
	n = 1814 (100%)	Yes n = 672 (37.0%)	No n = 1142 (63.0%)
Female, n (%)	959 (52.9)	284 (42.3)	675 (59.1)
Age, yrs, n (%)			
< 20	13 (0.7)	3 (0.4)	10 (0.9)
20–34	145 (8.0)	19 (2.8)	126 (11.0)
35–49	197 (10.9)	35 (5.2)	162 (14.2)
50–64	383 (21.1)	120 (17.9)	263 (23.0)
≥ 65	1076 (59.3)	495 (73.6)	581 (50.9)
Age, median yrs (IQR)	68 (55–79)	73 (64–81)	65 (49–76)
Anticoagulant or platelet inhibitor, n (%)*			
Any anticoagulant	396 (21.8)	396 (58.9)	–
LMWH therapeutic	95 (5.2)	95 (14.1)	–
LMWH prophylactic	214 (11.8)	214 (31.8)	–
Non-LMWH anticoagulants	102 (5.6)	102 (15.2)	–
Any platelet inhibitor	328 (18.1)	328 (48.8)	–
Non-ASA platelet inhibitor	68 (3.7)	68 (10.1)	–
ASA	304 (16.8)	304 (45.2)	–
Pathogen in urine, n (%)*			
Any Gram-negative	1579 (86.5)	567 (84.4)	1003 (87.8)
Any *E. coli*	1078 (59.4)	333 (49.6)	745 (65.2)
Any Gram-negative other than *E. coli*	547 (30.2)	254 (37.8)	293 (25.7)
Any Gram-positive	333 (18.4)	155 (23.1)	278 (15.6)
Any other pathogen than *E. coli* (both gram-negative and gram-positive)	834 (46.0)	383 (57.0)	451 (39.5)
≥ 2 pathogens in urine	165 (9.1)	82 (12.2)	83 (7.3)
Clinical presentation, n (%)*			
Fever	1806 (99.6)	671 (99.9)	1135 (99.4)
Urinary catheter	368 (20.3)	211 (31.4)	157 (13.7)
CRP levels, median mg/L (IQR)	84.5 (38–162)	84 (40–161)	85 (36–162)
WBC count, median count × 10.^9^/L (IQR)	11.6 (8.2–15.5)	12.0 (8.6–15.7)	11.4 (7.9–15.4)
Comorbidities, n (%)*			
Diabetes	295 (16.3)	148 (22.0)	147 (12.9)
Malignancy	583 (32.1)	231 (34.8)	352 (30.8)
Hypertension	521 (28.7)	287 (42.7)	234 (20.5)
Chronic Renal Failure	165 (9.1)	96 (14.3)	69 (6.0)
Chronic Liver Disease	25 (1.4)	6 (0.9)	19 (1.7)
Congestive Heart Failure	208 (11.5)	122 (18.2)	86 (7.5)
Tachycardia arrhythmia	263 (14.5)	160 (23.8)	103 (9.0)
Atrial fibrillation	249 (13.7)	153 (22.8)	96 (8.4)
Cardiovascular disease	320 (17.6)	229 (34.1)	91 (8.0)
Thromboembolism	128 (7.1)	83 (12.4)	45 (3.9)
Coagulopathy	71 (3.9)	19 (2.8)	52 (4.6)
BMI			
< 18.5 kg/m^2^	241 (13.3)	96 (14.3)	145 (12.7)
18.5–25 kg/m^2^	405 (22.3)	156 (23.2)	249 (21.8)
25–30 kg/m^2^	369 (20.3)	165 (24.6)	204 (17.9)
≥ 30 kg/m^2^	198 (10.9)	93 (13.8)	105 (9.2)
Missing	601 (33.1)	162 (24.1)	439 (38.4)

IQR: inter quartile range; LMWH: low molecular weight heparin; ASA: Acetylsalicylic acid; CRP: C-reactive protein; WBC: white blood cell; BMI: Body Mass Index

^*^Categories are potentially overlapping

### Bacteremia among patients with acute pyelonephritis

**Fig. 2:**
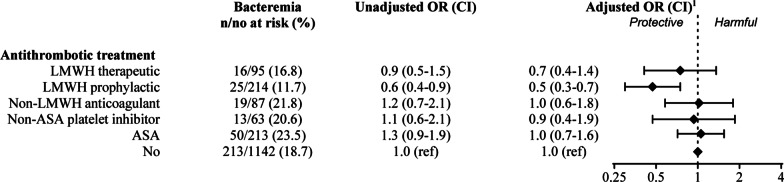
Odds ratio (OR) of bacteremia during acute pyelonephritis (n = 1814). Number of cases, unadjusted OR and adjusted OR as well as confidence intervals (CIs) are presented. CIs not spanning 1 are considered significant. LMWH: low molecular weight heparin; ASA: Acetylsalicylic acid. ^1^ adjusted by age, sex, BMI, malignancy, diabetes, hypertension, chronic renal failure, congestive heart failure, chronic liver disease, cardiovascular disease, thromboembolism and coagulopathy

In the first logistic regression analyses, we compared the risk of bacteremia in patients with different types of antithrombotic treatment to patients without such treatment. With the exception of a lower risk of bacteremia among patients treated with LMWH at prophylactic doses (adjusted OR 0.5, 95% CI 0.3–0.7), there were no differences in risk of bacteremia between the other treatments. The other treatments included LMWH at therapeutic doses (adjusted OR 0.7, 95% CI 0.4–1.4), non-LMWH anticoagulants (adjusted OR 1.0, 95% CI 0.6–1.8), non-acetylsalicylic acid platelet inhibitors (adjusted OR 0.9, 95% CI 0.4–1.9), and acetylsalicylic acid (adjusted OR 1.0, 95% CI 0.7–1.6) (Fig. 2).

### Acute kidney injury among patients with acute pyelonephritis

**Fig. 3:**
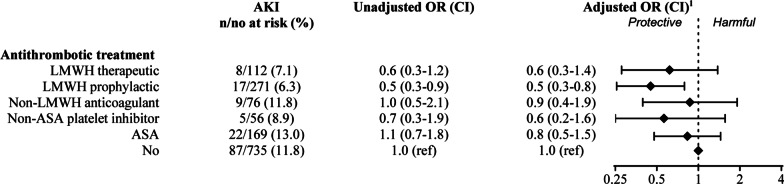
Odds ratio (OR) of acute kidney injury during acute pyelonephritis (n = 1419). Number of cases, unadjusted OR and adjusted OR as well as confidence intervals (CIs) are presented. CIs not spanning 1 are considered significant. LMWH: low molecular weight heparin; ASA: Acetylsalicylic acid. ^1^ adjusted by age, sex, BMI, malignancy, diabetes, hypertension, chronic renal failure, congestive heart failure, chronic liver disease, cardiovascular disease, thromboembolism and coagulopathy

1419 patients with acute pyelonephritis had sufficient data to allow ascertainment of renal function throughout the acute pyelonephritis episode. Of these, 148 (10.4%) developed acute kidney injury (Fig. 3). When fitted in a logistic regression model, patients treated with LMWH at prophylactic doses had statistically significantly lower risks of acute kidney injury compared to no antithrombotic treatment, with odds ratios of 0.5 (95% CI 0.3–0.8). There were no differences in risk of acute kidney injury between the other treatments and no antithrombotic treatment (Fig. 3).

## Discussion

In this study we tested a hypothesis that following bacterial infection of renal tubules, coagulation in local capillaries form a physical barrier that limits the systemic spread of bacteria. Antithrombotic drugs may inhibit this apparently protective process and thereby promote bacteremia. This hypothesis was inspired by our experimental studies in rats as well as reports exploring the initiation of coagulation during localized acute pyelonephritis [14, 17, 20–22]. In addition to hindering systemic bacterial spread, we speculated that this vascular disruption may also lead to more prominent kidney injury due to local ischemia. In contrast to our initial hypothesis, we did not find any convincing evidence of an increased risk of bacteremia in patients on antithrombotic treatment during acute pyelonephritis. Rather, we found a slightly lower risk of both bacteremia and acute kidney injury in patients undergoing treatment with LMWH at prophylactic doses, compared to patients without any antithrombotic treatment. As such, our results suggest that it is safe to continue ongoing antithrombotic treatment during acute pyelonephritis, with regards both to the risk of bacteremia and acute kidney injury.

To our knowledge, this is the first study on the association between antithrombotic treatment and bacteremia in patients with acute pyelonephritis. While there are several studies on anticoagulant therapy as adjunctive treatment of sepsis [7–12], studies investigating associations between anticoagulants or platelet inhibitors and the risk of bacteremia are scarce. A small number of studies have investigated an association between anticoagulant treatment and risk of bacteremia, but did not take the site of infection origin into consideration [23, 24]. A lower risk of bacteremia has been reported among patients with atrial fibrillation treated with dabigatran, compared to patients treated with factor Xa-inhibitor [23]. However, this particular study did not compare risks of bacteremia in patients with and without anticoagulant treatment, which was the focus of our study. In contrast to a study showing increased risk of intravenous catheter-related bacteremia among patients with systemic anticoagulant treatment [24], we found a slightly reduced risk of bacteremia among patients in our cohort with prophylactic doses of LMWH. However, the causative microorganisms and their mode of infection are very different between these two studies, which could explain our contradictory findings.

The anticoagulant used in our original animal studies was unfractioned heparin [14], which is less frequently used in clinical practice and was not prescribed to any patients included in our current study. However, 309 patients were treated with LMWH, among whom we saw a reduced risk of bacteremia. Together with our observation that there was no difference in risk of bacteremia among patients with therapeutic doses of LMWH or other antithrombotics, this suggests that patients with antithrombotic treatment do not have higher risk of bacteremia during acute pyelonephritis. Still, we are cautious to suggest any strong protective effect of LMWH at prophylactic doses.

While it is clinically comforting that we did not find any evidence of increased risk of bacteremia among patients with antithrombotic treatment, we did observe that treatment with prophylactic doses of LMWH reduced the risk of acute kidney injury. This is in line with recent studies showing that both unfractioned heparin and LMWH treatment prevent acute kidney injury during acute pyelonephritis and sepsis [25–28]. While our results partly support our hypothesis that activation of coagulation might contribute to acute kidney injury during acute pyelonephritis, the lack of convincing evidence of a protective effect of LMWH at therapeutic doses or non-LMWH anticoagulants, means we cannot draw any conclusions regarding the underlying mechanisms of these findings. The multifaceted biological effects of heparins, including both anticoagulatory [29] and immunomodulatory effects [30–34], complicate the interpretation of both clinical and experimental studies using heparin or LMWH. However, we believe that our results highlight a potentially important association for further study.

The strengths of our study include a relatively large sample size and our focus on a group of patients who are at a potentially higher risk of bacteremia. We also included and adjusted for a range of clinically relevant covariates and potential confounders, with little or no missing data. Despite the strengths, there are of course limitations to consider. First, it is a single-center, observational study, and therefore generalization of our results should be interpreted cautiously. Being the first study of its kind, however, our work motivates further larger-scale prospective studies to validate these results. Second, there are restrictions in the available data. Due to the retrospective nature of the study, we did not have access to structured data for each hospital stay. For example, we do not know the exact duration of symptoms for each patient, and they were therefore likely included in the study at different stages of infection. Patients with antithrombotic treatment may also have different comorbidity burdens, and therefore have a different propensity to seek medical care. Thus, patients with and without antithrombotic treatment might have different baseline risks of bacteremia, as a longer duration of symptoms prior to diagnosis and initiation of antibiotic treatment, is related to higher risk of bacteremia and sepsis [35]. Further, due to the presence of certain comorbidities clinicians may be more inclined to admit patients with antithrombotic treatment to the hospital or to start antibiotic treatment earlier or with intravenous antibiotics. These effects are likely not fully removed by adjustment for comorbidity and other patient factors. Patients on anticoagulants may also have been kept in the hospital long enough to follow up with further serum creatinine measurements, allowing for detection of acute kidney injury. Therefore, missing data from patients without antithrombotic treatment may have resulted in the introduction of bias. Moreover, we did not have conclusive data on indications for antithrombotic treatment and could therefore not adjust for these as possible confounders. Thirdly, we only include patients treated in a university hospital setting, and only where blood cultures were obtained, which might limit generalizability. However, in this study we deliberately chose to observe a patient group with reported higher risk of bacteremia and where the occurrence of the outcome could be accurately ascertained. Further, our results show that bacteremia was present in approximately 20% of the cases, which is in line with earlier findings [1, 36, 37].

## Conclusions

Patients with acute pyelonephritis and antithrombotic treatment do not appear to have a higher risk of developing bacteremia. In contrast to our initial hypothesis, we found a slightly lower risk of bacteremia among patients with LMWH treatment at prophylactic doses. For acute kidney injury, concordant with our hypothesis, we found that treatment with LMWH at prophylactic doses also seem to have protective effects. The observed decreases need to be confirmed in future studies. Collectively, our findings suggest that it is safe to continue antithrombotic treatments in patients with acute pyelonephritis if there are no other contraindications. While our results might indicate that coagulation has a role during acute pyelonephritis, future studies validating the potential protective effects, and investigating possible causative relationships are needed.

## Supplementary Information

**Additional file 1: Table S1. **Antithrombotic drugs included in the analyses. **Table S2. **Definitions of disease, co-morbidities, and outcomes. **Figure S1. **Flowchart of the study population and cohort studied in sub-analysis. **Table S3.** Bacterial strains found in urine cultures of 1814 patients. **Table S4.** Bacterial strains found in positive blood cultures of 336 patients (18.5%) out of a total of 1814 patients.

## Data Availability

Individual level data cannot be shared publicly because of patient confidentiality under current Swedish legislation. The data that support the findings of this study are available from Karolinska University Hospital but restrictions apply to the availability of these data, which were used under license for the current study, and so are not publicly available. Data are however available from the authors upon reasonable request and with permission of Karolinska University Hospital and the appropriate ethics committee.

## Abbreviations

ATC: Anatomic Therapeutic Chemical (ATC) classification codes
ASA: Acetylsalicylic acid
BMI: Body mass index
CI: Confidence interval
DOAC: Direct oral anticoagulants
GCS: Glasgow Coma Scale
IQR: Inter quartile range
LMWH: Low-molecular-weight heparin
OR: Odds ratio
