# Supplementary material for: Effect of anticoagulant and platelet inhibition on the risk of bacteremia among patients with acute pyelonephritis: a retrospective cohort study
Source: BMC Infect Dis. 2022 May 31;22:509. doi: 10.1186/s12879-022-07474-4 (PMC9158213; doi:10.1186/s12879-022-07474-4)
Supplement: Supplementary file 1 — Additional file 1: Table S1. Antithrombotic drugs included in the analyses. Table S2. Definitions of disease, co-morbidities, and outcomes. Figure S1. Flowchart of the study population and cohort studied in sub-analysis. Table S3. Bacterial strains found in urine cultures of 1814 patients. Table S4. Bacterial strains found in positive blood cultures of 336 patients (18.5%) out of a total of 1814 patients. [file 12879_2022_7474_MOESM1_ESM.docx]

**Additional file 1: Supplementary material**

**Effect of anticoagulant and platelet inhibition on the risk of bacteremia among patients with acute pyelonephritis: a retrospective cohort study**

Svava E. Steiner, Gustaf Edgren, Keira Melican, Agneta Richter-Dahlfors, Annelie Brauner

**Contents:**

**Table S1.** Antithrombotic drugs included in the analyses.

**Table S2.** Definitions of disease, co-morbidities, and outcomes.

**Figure S1.** Flowchart of the study population and cohort studied in sub-analysis.

**Table S3.** Bacterial strains found in urine cultures of 1814 patients.

**Table S4.** Bacterial strains found in positive blood cultures of 336 patients (18.5%) out of a total of 1814 patients.

| **ATC-code** | **Drug name** | **Type** | **n** | **%** |
| --- | --- | --- | --- | --- |
|  |  |  |  |  |
| B01AB04 | Dalteparin | LMWH | 267 | 33,5 |
| B01AB05 | Enoxaparin | LMWH | 19 | 2,4 |
| B01AB10 | Tinzaparin | LMWH | 34 | 4,3 |
| B01AA03 | Warfarin | Vitamin-K antagonist | 67 | 8,4 |
| B01AE07 | Dabigatran | DOAC | 5 | 0,6 |
| B01AF01 | Rivaroxaban | DOAC | 6 | 0,8 |
| B01AF02 | Apixaban | DOAC | 25 | 3,1 |
| B01AC04 | Clopidogrel | Platelet inhibitor | 40 | 5,0 |
| B01AC06 | ASA | Platelet inhibitor | 304 | 38,2 |
| B01AC07 | Dipyridamole | Platelet inhibitor | 19 | 2,4 |
| B01AC11 | Iloprost | Platelet inhibitor | 1 | 0,1 |
| B01AC22 | Prasugrel | Platelet inhibitor | 9 | 1,1 |
| B01AC24 | Ticagrelor | Platelet inhibitor | 267 | 33,5 |
|  |  |  |  |  |
|  |  |  | 796 | 100% |
|  |  |  |  |  |

**Table S1.** Antithrombotic drugs included in the analyses.

Abbreviations: ATC-code=Anatomic Therapeutic Chemical classification system, LMWH=low-molecular-weight heparin, DOAC=direct oral anticoagulant, ASA=Acetylsalicylic acid. Some patients were prescribed more than one antithrombotic treatment.

**Table S2.** Definitions of disease, co-morbidities, and outcomes.

| **Variable** | **Definition** |
| --- | --- |
| Fever | Measured body temperature ≥38^o^C  or registered diagnosis of fever (ICD-10 R50.8 and R50.9) |
| Pyelonephritis | Positive urine culture and registered fever (see definition above)  or registered diagnosis of acute pyelonephritis (ICD-10 N10.9, N12.9, N15.1) |
|  |  |
| Age categories | Age was categorized into 4 categories based on the age quartiles of the cohort (1^st^ quartile 18-56 years, 2^nd^ quartile 57-69 years, 3^rd^ quartile 70-78 years, 4^th^ quartile 79-101 years) |
|  |  |
| BMI categories | BMI was categorized into the following 5 categories: “BMI < 18.5”, “BMI 18.5-24.9”, “BMI 25-30”, “BMI >30”, and “BMI missing” |
|  |  |
| **Anticoagulant treatment** |  |
| LMWH | ATC-code: B01AB04=Dalteparin, B01AB05=Enoxaparin, B01AB10=Tinzaparin |
| Therapeutic doses | Daily dose of more than 5000 units Dalteparin, more than 4000 units Enoxaparin or more than 4500 units Tinzaparin. |
| Prophylactic doses | Daily dose of up to 5000 units Dalteparin, 4000 units of Enoxaparin or 4500 units Tinzaparin. |
|  |  |
| Non-LMWH anticoagulant | ATC-code: B01AA03=Warfarine, B01AE07=Dabigatran (DOAC), B01AF01=Rivaroxaban (DOAC), B01AF02=Apixaban (DOAC). |
|  |  |
| Non-ASA platelet inhibitor | Any ATC-code starting with “B01AC”, except B01AC06 |
|  |  |
| ASA | ATC-code: B01AC06 |
|  |  |
| **Co-morbidities** |  |
| Diabetes | ICD-10: E10, E11 |
| Malignancy | ICD-10: C00-C09, C10-C19, C20-C26, C30-C39, C41-C43, C45-C49, C50-C58, C60-C69, C70-C79, C80-C88, C90-C97, Z85 |
| Hypertension | ICD-10: I10-I15 |
| Chronic Renal Failure | ICD-10: I12.0, N18 |
| Chronic Liver Disease | ICD-10: K70, K72.1, K72.9, K74.6, K76.9 |
| Congestive Heart Failure | ICD-10: I11.0, I50 |
| Tachycardia arrhythmia | ICD-10: I45.6, I47, I48, R00.0 |
| Atrial fibrillation | ICD-10: I48 |
| Cardiovascular disease | ICD-10: I20-I25, I63-I67, I69, I70, I73.9, Z86.6A, Z86.7 |
| Thromboembolism | ICD-10: I26, I74, I81, I82, Z86.7A, Z86.7B |
| Coagulopathy | ICD-10: D65, D66, D68, D69 |
|  |  |
| **Outcomes** |  |
| Bacteremia | Growth of the same pathogen in blood as in urine |
| Acute kidney injury | ICD-10: N17 or R34.9  or 50% increase or 26.4 μmol/l increase in serum creatinine levels within 48 h of the first positive urine culture, compared to baseline serum creatinine levels |
|  |  |

Abbreviations: ICD-10 = International Statistical Classification of Diseases and Related Health Problems – 10th Revision, ATC-code=Anatomic Therapeutic Chemical classification system, LMWH=low-molecular-weight heparin, DOAC=direct oral anticoagulant, ASA=Acetylsalicylic acid, eGFR = estimated Glomerular Filtration Rate.


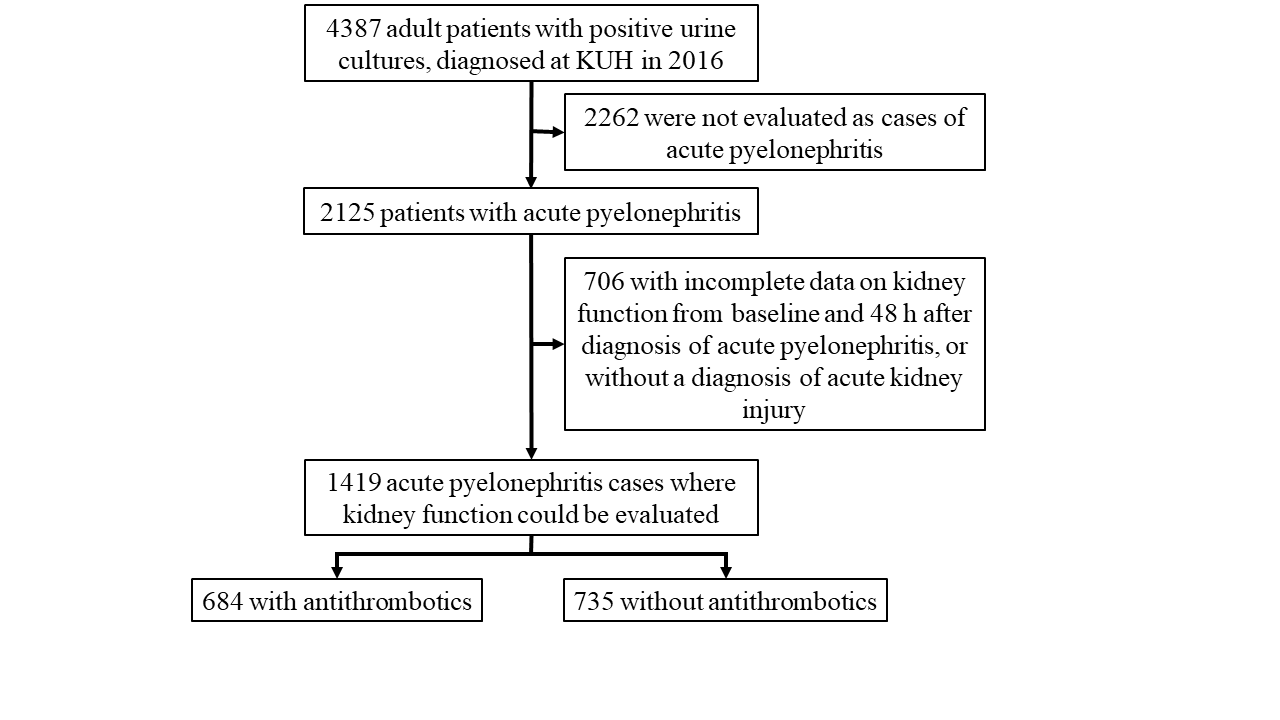


**Figure S1.** Flowchart of the study population and cohort studied in sub-analysis. Abbreviations: KUH = Karolinska University Hospital.

**Table S3.** Bacterial strains found in urine cultures of 1814 patients.

|  | **Pathogen** | |  | **n*** |  | **(%)** |  |
| --- | --- | --- | --- | --- | --- | --- | --- |
|  |  | |  |  |  |  |  |
|  | **Gram-negative** | |  |  |  |  |  |
|  | | *Acinetobacter baumannii* |  | 3 |  | 0,2 |  |
|  | | *Aeromonas species* |  | 1 |  | 0,1 |  |
|  | | *Citrobacter amalonaticus* |  | 1 |  | 0,1 |  |
|  | | *Citrobacter freundii* |  | 16 |  | 0,8 |  |
|  | | *Citrobacter koseri* |  | 25 |  | 1,3 |  |
|  | | *Citrobacter species* |  | 2 |  | 0,1 |  |
|  | | *Enterobacter aerogenes* |  | 20 |  | 1,0 |  |
|  | | *Enterobacter cloacae* |  | 46 |  | 2,3 |  |
|  | | *Escherichia coli* |  | 1078 |  | 54,3 |  |
|  | | Gram-negative rod, *Enterobacteriaceae* |  | 3 |  | 0,2 |  |
|  | | *Haemophilus parainfluenzae* |  | 2 |  | 0,1 |  |
|  | | *Hafnia alvei* |  | 1 |  | 0,1 |  |
|  | | *Klebsiella oxytoca* |  | 65 |  | 3,3 |  |
|  | | *Klebsiella pneumoniae* |  | 192 |  | 9,7 |  |
|  | | *Klebsiella species* |  | 1 |  | 0,1 |  |
|  | | *Morganella morganii* |  | 16 |  | 0,8 |  |
|  | | *Pantoea species* |  | 2 |  | 0,1 |  |
|  | | *Proteus mirabilis* |  | 65 |  | 3,3 |  |
|  | | *Proteus vulgaris* |  | 2 |  | 0,1 |  |
|  | | *Providencia species* |  | 2 |  | 0,1 |  |
|  | | *Pseudomonas aeruginosa* |  | 79 |  | 4,0 |  |
|  | | *Pseudomonas species* |  | 11 |  | 0,6 |  |
|  | | *Serratia marcescens* |  | 10 |  | 0,5 |  |
|  | | *Stenotrophomonas maltophilia* |  | 4 |  | 0,2 |  |
|  | |  |  |  |  |  |  |
|  | | **Gram-positive** |  |  |  |  |  |
|  | | Alpha-hemolytic *Streptococcus* |  | 4 |  | 0,2 |  |
|  | | Beta-hemolytic *Streptococcus* group A |  | 7 |  | 0,4 |  |
|  | | Beta-hemolytic *Streptococcus* group C |  | 1 |  | 0,1 |  |
|  | | Beta-hemolytic *Streptococcus* group G |  | 11 |  | 0,6 |  |
|  | | *Enterococcus faecalis* |  | 80 |  | 4,0 |  |
|  | | *Enterococcus faecium* |  | 49 |  | 2,5 |  |
|  | | *Enterococcus gallinarum* |  | 2 |  | 0,1 |  |
|  | | *Enterococcus species* |  | 150 |  | 7,6 |  |
|  | | *Streptococcus anginosus (milleri)* |  | 6 |  | 0,3 |  |
|  | | *Streptococcus*  Group B *(Streptococcus agalactiae)* |  | 26 |  | 1,3 |  |
|  | | *Streptococcus species* |  | 1 |  | 0,1 |  |
|  |  | |  |  |  |  |  |
|  | Total | |  | 1984 |  | 100.0 |  |
|  |  | |  |  |  |  |  |
| *For some patients more than one bacterial strain was found in urine. | | | | | | | |

**Table S4.** Bacterial strains found in positive blood cultures of 336 patients (18.5%) out of a total of 1814 patients.

|  | **Pathogen** |  | **n*** |  | **(%)** |  |
| --- | --- | --- | --- | --- | --- | --- |
|  |  |  |  |  |  |  |
|  | **Gram-negative** |  |  |  |  |  |
|  | *Citrobacter koseri* |  | 5 |  | 1,5 |  |
|  | *Enterobacter aerogenes* |  | 3 |  | 0,9 |  |
|  | *Enterobacter cloacae* |  | 5 |  | 1,5 |  |
|  | *Escherichia coli* |  | 221 |  | 64,2 |  |
|  | Gram-negative rod |  | 1 |  | 0,3 |  |
|  | *Klebsiella oxytoca* |  | 9 |  | 2,6 |  |
|  | *Klebsiella pneumoniae* |  | 41 |  | 11,9 |  |
|  | *Klebsiella species* |  | 1 |  | 0,3 |  |
|  | *Morganella morganii* |  | 2 |  | 0,6 |  |
|  | *Pantoea species* |  | 1 |  | 0,3 |  |
|  | *Proteus mirabilis* |  | 15 |  | 4,4 |  |
|  | *Proteus vulgaris* |  | 1 |  | 0,3 |  |
|  | *Pseudomonas aeruginosa* |  | 13 |  | 3,8 |  |
|  |  |  |  |  |  |  |
|  | **Gram-positive** |  |  |  |  |  |
|  | Alpha-hemolytic *Streptococcus* |  | 1 |  | 0,3 |  |
|  | Beta-hemolytic *Streptococcus* group A |  | 1 |  | 0,3 |  |
|  | Beta-hemolytic *Streptococcus* group G |  | 1 |  | 0,3 |  |
|  | *Enterococcus faecalis* |  | 16 |  | 4,7 |  |
|  | *Enterococcus faecium* |  | 6 |  | 1,7 |  |
|  | *Streptococcus anginosus (milleri)* |  | 1 |  | 0,3 |  |
|  |  |  |  |  |  |  |
|  | Total |  | 344 |  | 100.0 |  |
|  |  |  |  |  |  |  |

| *For some patients more than one bacterial strain was found in blood. |
| --- |
